# New Drugs and Praparations

**Published:** 1894-05-12

**Authors:** 


### NEW DRUGS AND PREPARATIONS.

[We shall be glad to receive, at our Office, 428, Strand, London, W.C., from the manufacturers, specimens of all new preparations and drugs which may be brought out from time to time.]

### APOCODEINE.

Guinard (*Lyon Médical*, 1893, Nos. 29—32) has shown that pure apocodeine has no emetic action. This is contrary to present received opinion, but he holds that previous investigators have been misled by the presence of apomorphine as an impurity. Its action on the whole resembles that of codeine. Experiments on dogs showed that small doses have a calming, hypnotic effect of moderate duration, and not followed by digestive disturbances. Larger doses tend to produce excitement and convulsions. There is transient stimulation of the circulation and respiration after small doses, followed by slowing during the period of sleep. Saliva, bile, and the secretions generally are increased, as well as that of the mucous glands. Peristalsis is also increased, and the pupil is not affected unless convulsions are induced, when it dilates. Compared with codeine, he found the latter less calmative and more convulsant, and therefore recommends apocodeine in its stead in all cases in which codeine is at present employed.

### CODEINE POISONING.

Dr. Spratling[1] records a case of poisoning from eight grains of codeine in a young married woman. It was taken at seven p.m. with the view of procuring sleep, and an hour later she vomited a small quantity of liquid matter. It is therefore uncertain how much was actually absorbed. Three hours after taking it she was awake, quite sensible, but very irritable and restless, then occurred convulsive movements, and great irritation of the skin, the pupils were pin-point, respirations 12 per minute, and thirst and fulness of the head were complained of. She was "subjected to the usual treatment for opium-poisoning, and in a few hours was much improved." By noon the following day she showed no ill-effects except considerable muscular weakness.

[1] Med. Record, July 15, 1893.

